# Usefulness of Ki-67, Mitoses, and Tumor Size for Predicting Metastasis in Carcinoid Tumors of the Lung: A Study of 48 Cases at a Tertiary Care Centre in Canada

**DOI:** 10.1155/2015/545601

**Published:** 2015-12-06

**Authors:** M. G. Joseph, A. Shibani, N. Panjwani, A. Arab, J. Shepherd, L. W. Stitt, R. Inculet

**Affiliations:** ^1^Department of Pathology, London Health Sciences Centre, Western University, London, ON, Canada; ^2^Department of Pathology, University Hospital, 339 Windermere Road, London, ON, Canada N6A 5A5; ^3^Brantford General Hospital, ON, Canada; ^4^Pathologist assistant program, London Health Sciences Centre, Western University, Canada; ^5^University of Calgary, Canada; ^6^Division of Respirology, University of Ottawa, Canada; ^7^Department of Thoracic Surgery, London Health Sciences Centre, Western University, London, ON, Canada

## Abstract

*Background*. Evaluation of Ki-67 index in lung carcinoid tumors (LCTs) has been of interest in order to identify high risk subsets. Our objectives are (1) to evaluate the usefulness of Ki-67 index, mitoses, and tumor size in predicting metastasis and (2) to compare the Manual Conventional Method (MCM) and the Computer Assisted Image Analysis Method (CIAM) for Ki-67 calculation.* Methods*. We studied 48 patients with LCTs from two academic centres in Canada. For Ki-67 calculation, digital images of 5000 cells were counted using an image processing software and 2000 cells by MCM. Mitoses/10 HPF was counted.* Results*. We had 37 typical carcinoids (TCs) and 11 atypical carcinoids (ACs). 7/48 patients developed metastasis. There was a positive relationship between metastasis and carcinoid type (*P* = 0.039) and metastasis and mitoses (≥2) (*P* = 0.017). Although not statistically significant, the mean Ki-67 index for ACs was higher than for TCs (0.95% versus 0.72%, CIAM, *P* = 0.299). Similarly, although not statistically significant, the mean Ki-67 index for metastatic group (MG) was higher than for nonmetastatic group (NMG) (1.01% versus 0.71% by CIAM, *P* = 0.281). However when Ki-67 index data was categorized at various levels, there is suggestion of a useful cutoff (≥0.50%) to predict metastasis (*P* = 0.106, CIAM). A significantly higher proportion of patients with mitosis ≥2 and Ki-67 index ≥0.50% had metastasis (*P* = 0.033) compared to other patients. Similarly patients with tumor size ≥3 cm and Ki-67 ≥0.50% had a greater percentage of metastases than others (*P* = 0.039). Although there was a strong correlation between two (MCM versus CIAM) counting methods (*r* = 0.929, *P* = 0.001), overall the calculated Ki-67 index was slightly higher by MCM (range 0 to 6.4, mean 1.5) compared to CIAM (range 0 to 2.9, mean 0.75).* Conclusion*. This study confirms that mitoses ≥2 is a powerful predictor of metastasis in LCTs. Although this is a small sample size, there is suggestion that analysis of Ki-67 index along with mitoses and tumor size may be a useful adjunct for predicting metastasis in LCTs.

## 1. Introduction

Carcinoid is a relatively uncommon neuroendocrine tumor of the lung. These tumors are classified as typical carcinoids (TC) and atypical carcinoids (AC) using Travis criteria who defined TC as a tumor with <2 mitoses/10 HPF and no necrosis, whereas the presence of spotty necrosis or 2–10 mitoses/10 HPF are diagnostic of AC [1, 2].

Traditional staging and histologic grading have been used to predict the biologic behavior of LCTs. Recently there is much interest to evaluate proliferation markers particularly Ki-67 in neuroendocrine tumors arising from various sites in an attempt to identify high risk subsets that may behave more aggressively. At a 2009 European Neuroendocrine Society Consensus Conference, an international group of experts proposed a grading system (G1–G3) utilizing a combination of Ki-67 and mitoses to stratify patients with GI neuroendocrine tumours [3]. However, there is only limited information in the literature on LCTs correlating mitotic rate, Ki-67 index, and clinical outcome.

In this study our objectives are (1) to evaluate the usefulness of Ki-67 index as well as traditional parameters such as mitoses and tumor size for predicting metastasis in LCTs and (2) to develop a systematic counting method for Ki-67 estimation in LCTs and compare the Manual Conventional Method (MCM) and the Computer Assisted Image Analysis Method (CIAM) for calculating Ki-67 index. We hypothesis that in LCTs in addition to mitoses, Ki-67 index might be an important parameter for identifying high risk subsets of patients for whom multimodal therapy may be considered for management.

## 2. Materials and Methods

### 2.1. Histologic Analysis

We conducted a computerized search of the pathology records at London Health Sciences Centre (LHSC) and St. Joseph's Health Centre (SJHC) in London, Ontario, Canada, over a 25-year period (1982–2007). Forty-eight consecutive patients who had a histologic diagnosis of LCTs in resected specimens were selected for this study. All cases were reclassified into TC and AC using the criteria by Travis et al. [1]. Slides were reviewed and mitoses were counted using a Zeiss microscope at 40x objective in three sets of 10 HPF (6 mm^2^ of viable tumor), and the average mitotic figure per 10 HPF (2 mm^2^) was calculated as suggested by Travis et al. [1]. Patient demographics and tumor characteristics as well as metastatic and survival data were evaluated by chart review. Two representative sections of tumor were stained for Ki-67 (Vector laboratories; antibody to Ki-67 antigen, clone MM1, Burlingame, CA, USA) by ABC method (heat-induced epitope retrieval with citrate buffer, prediluted (1 : 200), completed manually at LHSC immunohistochemistry lab). Ki-67 counting was performed using both Computer Assisted Image Analysis Method (CIAM) and a Manual Conventional Method (MCM). The Ki-67 labeling index was calculated as the ratio of number of stained cells to total number of cells expressed as a percentage. Both mitotic count and Ki-67 counting were performed in a blind fashion without knowledge of clinical data.

### 2.2. Ki-67 Counting by Computer-Assisted Image Analysis Method

**Figure 1:**
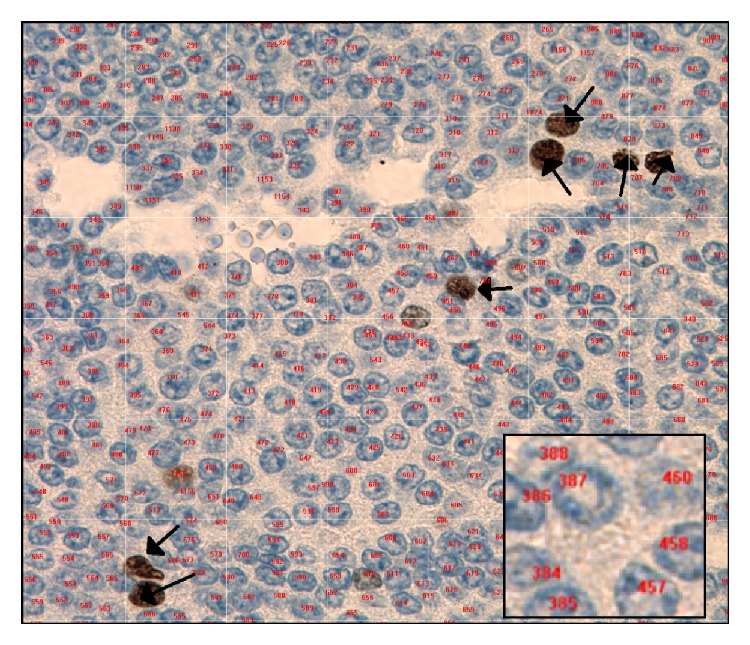
Counting of Ki-67 positive cells by computer-assisted image analyzer method. The positive cells are dark brown and indicated by black arrows. The negative cells are blue and numbered in red (inset).

For quantitative evaluation of Ki-67 staining, densely stained areas from tumor were systematically identified on immunostained sections at high power field (wide field 10x oculars and 60x objective, Olympus BX51/52 System Microscope; Melville, NY, USA). Up to ten high power fields were selected from these high density areas for independent evaluation and a minimum total of 5000 tumor cells including the positive stained cells were counted for each case by one of the authors (N. Panjwani) to calculate the Ki-67 index. The number 5000 tumor cells was chosen based on statistical analysis by calculating the optimal number of cells to be counted based on 95% confidence interval (CI) using multiple proportion of cells. Five thousand cells proved to be the optimal number with a relatively tighter CI. Images were digitized as TIF images using a digital camera system (Pursuit Slider, SPOT Diagnostic Instruments Inc.; Sterling Heights, MI, USA) and imported into a Microcad PC Desktop Computer (Model ANT 303x). The stored digital images were analyzed with image-processing software (Northern Eclipse Version 7.0, Empix Imaging Inc.; Mississauga, ON, Canada) with a superimposed grid and manual count tool options. Positive staining was observed as dark brown staining of the nuclei in tumor cells (Figure 1). Lymphocytes and endothelial cells were excluded from counting.

### 2.3. Ki-67 Counting by Manual Conventional Method

Slides were scanned in a routine manner and areas of highest density staining were located. Using a Zeiss microscope and 40x objective one author (A. Shibani) manually counted a minimum of 2000 tumor cells to calculate the Ki-67 labeling index as suggested by Rindi et al. [3]. Positive nuclear staining of tumor cells under the microscope was of varying intensity, mostly moderate to strong and some mild, and any staining was considered as positive staining.

### 2.4. Statistical Analysis

The development of metastasis was considered as the end outcome point in our study and therefore we divided the patients into these 2 groups, metastatic (MG) and nonmetastatic (NMG) groups. Between-group comparisons were made using Fisher's exact two-tailed tests. The relationship between Ki-67 indices and carcinoid subtypes was analyzed with an unpaired *t* test for independent variables. Ki-67, mitoses, and tumour size were categorized at various levels. Clinically meaningful combinations of mitoses and Ki-67, size and Ki-67, and mitoses and tumor size were evaluated. For MG and NMG sensitivity, specificity and odds ratios and their 95% confidence intervals were calculated. To evaluate the relationship between the computerized and manual counting methods, statistical analysis was completed using the Pearson Correlation Coefficient. A *P* value < 0.05 is considered as statistically significant, and *r* value > 0.8 is considered as strong correlation.

## 3. Results

The age of the patients ranged from 17 to 81 years with a mean age of 52 years. Eighteen patients were male and 30 patients were female. All patients underwent surgical resection as primary treatment (38 lobectomy, 8 pneumonectomy, and 2 wedge resection). Out of 48 cases, 37 were classified as TCs and 11 ACs. Patient follow-up ranged from 0.5 months to 306.8 months, with median follow-up of 45 for metastatic group (MG) and 35 for nonmetastatic group (NMG). The tumor size ranged from 0.5 cm to 9.5 cm (mean 2.7 cm) in greatest diameter. The mitoses ranged from 0 to 11.6 (mean 2.7). Seven out of 48 patients developed metastasis: 6 in mediastinal lymph nodes, 3 in liver, and 2 in both. Lymphovascular invasion was identified in 4 cases, 3 of which were TCs. Six patients presented with endocrine symptoms: carcinoid syndrome [4] and Cushing syndrome [1]. Four patients with metastatic disease received adjuvant therapy: chemotherapy [2] and radiotherapy [2]. Three patients died, one as a result of stroke and two from liver metastasis.

Table 1 shows the relationship between carcinoid type, metastasis, and Ki-67 index calculated by both counting methods. Although not statistically significant, the mean Ki-67 index for atypical carcinoids was higher (0.95% versus 0.72%, CIAM, *P* = 0.299; 2.32% versus 1.37%, MCM, *P* = 0.71) than for typical carcinoid by both counting methods. Similarly, when we analyzed the relationship between Ki-67 index and metastasis, although not statistically significant, the mean Ki-67 index for MG was higher than for NMG (1.01% versus 0.71%, CIAM, *P* = 0.281; 2.10% versus 1.39%, MCM, *P* = 0.239).  Table 2 shows the relationship between various factors (carcinoid type, size, and mitosis and Ki-67 index) and metastasis at various levels. The sensitivity, specificity, odd ratio, and *P* value were calculated. As expected there is a statistically significant correlation between metastasis and carcinoid type (*P* = 0.039) and mitoses ≥2 (*P* = 0.017) with relatively high specificity. In addition, when Ki-67 index data was categorized at various levels, although not statistically significant, there was suggestion of a useful cutoff (≥0.50%) to predict metastasis by both counting methods with relatively high sensitivity. Similarly although not statistically significant there was suggestion of a useful cutoff for tumor size (≥3 cm) to predict metastasis with relatively high sensitivity and specificity.

We performed similar statistical analysis combining multiple clinical and pathologic factors (Table 2). A significantly higher proportion of patients with mitosis ≥2 and Ki-67 index ≥0.50% had metastasis (*P* = 0.033) with high specificity. Similarly patients with tumor size ≥3 cm and Ki-6 7 ≥ 0.50% had a greater percentage of metastases (*P* = 0.039) with high specificity. In this study, there was no statistically significant correlation between metastatic disease and patient age, sex, presence or absence of lymphovascular invasion, endocrine symptoms, or adjuvant therapy.

**Figure 2:**
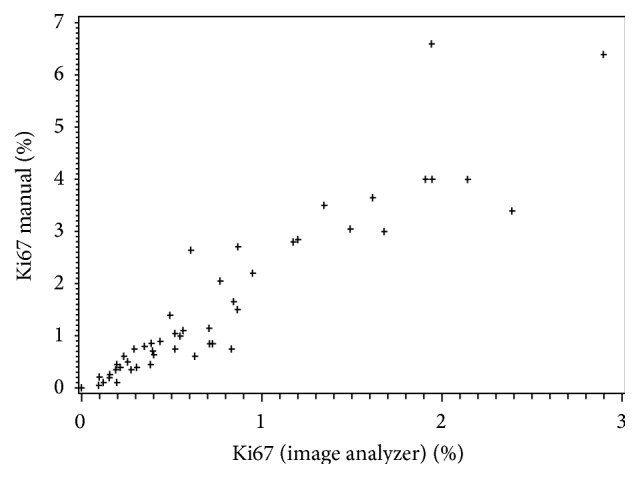
This plot diagram illustrates a linear relationship between computer image analysis and manual method for calculating Ki-67 index.

We used both MCM and CIAM for counting Ki-67. Although there was a linear relationship with strong correlation between these two counting methods (*r* = 0.929, *P* = 0.001) (Figure 2), overall the calculated Ki-67 index was higher by MCM (range 0 to 6.4, mean 1.5) than by CIAM (range 0 to 2.9, mean 0.75) (Table 3).

## 4. Discussion

Pulmonary neuroendocrine (NE) tumors encompass a spectrum with four tumour categories being identified by morphology, namely, low-grade TC, intermediate-grade AC, and high-grade large-cell neuroendocrine carcinoma (LCNEC) and small-cell lung carcinoma (SCLC) [4, 5]. The distinction between these tumors is critical because of significant difference in clinical behavior, therapy, and prognosis [1, 2, 6]. Carcinoid tumors are primarily treated by surgical resection and it is generally felt that, once advanced, nonresectable or metastasized patients may require chemotherapy, although no clear guidelines have been set for chemotherapy regimens.

Traditionally the biological behavior of carcinoid tumors is predicted by conventional methods such as staging and histologic grading using Travis criteria. Travis et al. [1] in a study of 200 pulmonary neuroendocrine tumors, of which 113 were carcinoids, have shown that patients diagnosed with typical carcinoids had a 5-year survival of 87% and 10-year survival of 87%. However, those diagnosed with atypical carcinoids had a drastically shorter 5-year survival of 56% and a 10-year survival of 35%.

During the past 10–15 years, newer biological markers have been sought to subtype carcinoid tumors and to identify high risk subtypes. Immunohistochemical analysis of the well-established proliferation marker Ki67 (MIB1) has received much attention and has been incorporated into a number of studies on pulmonary and extrapulmonary tumors. This nuclear antigen is a cell cycle associated protein that is expressed throughout the cell cycle in proliferating cells (G1-M) but not in resting (G0) cells [7]. It has been shown that Ki-67 reactivity significantly correlates with histological grading and proliferative activity and has been considered as a good marker for estimating tumor progression in a variety of nonpulmonary malignancies [8–11].

At a European Neuroendocrine Society Consensus Conference [3], an international group of experts proposed a grading system (G1–G3) of prognostic significance for digestive tract neuroendocrine neoplasms and it is incorporated into the 2010 World Health organization (WHO) classification. This grading system was based on mitotic count and proliferation marker analysis using Ki-67 index expressed in % positivity. This grading system has also been used by some medical oncologists to stratify patients with gastrointestinal neuroendocrine tumours for adjuvant chemotherapy.

In this study of 48 cases when we assessed the relationship between carcinoid subtype and metastatic disease, we noticed that 36% of patients with AC had metastasis compared to only 8% of patients with TC. This result emphasizes the observation of others that AC of lung is associated with worse prognosis than TC [1, 2, 12]. In addition we confirmed the observation of Travis that mitosis ≥2 is a powerful predictor of metastasis [1]. We also observed that Ki-67 index is quite low (mean 1.5% by MCM and 0.75% by CIAM) in LCTs and that there is a slightly higher Ki-67 index in atypical carcinoids compared to typical carcinoids, although not statistically significant (Table 1). We also observed that a higher proportion of patients who developed metastasis exhibited a Ki-67 index of ≥0.50% although not statistically significant. When we focused our attention on studying the relationships between multiple factors and development of metastasis, we noted that a significantly higher proportion of patients with mitoses ≥2 and Ki-67 index (by CAIM) ≥0.50% had metastasis. Similarly significantly higher proportion of patients with mitoses ≥2 and tumor size ≥3 cm had metastasis. Based on these observations, our study suggests that a cutoff of Ki-67 index (≥0.50%) along with mitoses ≥2 as well as Ki-67 index (≥0.50%) along with tumor size ≥3 cm may be useful in predicting metastatic potential of LCTs. Although the limited number of our patients makes it hard to draw an absolute conclusion, this cutoff needs to be tested on larger number of patients prospectively in future studies.

In this study we did not observe a statistically significant association between metastasis and a number of other clinically important factors such as age, sex, lymph vascular invasion, endocrine symptoms, adjuvant therapy, and death. However, it is important to note that the majority of patients who developed endocrine manifestations (6 patients) are those with a Ki-67 index >0.50% (6/6 by MCM, and 4/6 by CAIM). Granberg et al. [9] in a study of 43 typical bronchial carcinoids noted that patients presenting with endocrine symptoms had higher Ki-67 index (*P* = 0.02) than patients without endocrine symptoms at diagnosis.

When we reviewed the literature on Ki-67 index and pulmonary neuroendocrine [9, 10, 12–22] and selected examples of extrapulmonary neuroendocrine [8, 23–29] tumors, we observed that the total number of cells counted and the counting methods used by various authors are quite variable. However it is important to note that Warth et al. [22] in an interobserver study using 9 experienced pulmonary pathologists and 20 carcinoid tumours provided evidence that assessment of Ki-67 in pulmonary carcinoids results in much higher interobserver agreement compared to mitotic counting. Select studies addressing Ki-67 index in lung carcinoid tumours in which authors utilized Travis criteria are summarized in (Table 4). Costes et al. [12] in a study of 47 resected lung carcinoid tumors (31 TCs and 16 ACs) using a computer assisted image processor reported that, compared to typical carcinoids, atypical carcinoids have a higher expression of Ki-67 labeling expressed in percentage of stained nuclear surface relative to the total nuclear surface area (mean 0.45% versus 2.45%  *P* = 0.0035). The authors concluded that, by using a ≥4% cutoff, there was a significant difference in the 5-year overall survival rate of patients with pulmonary carcinoids and suggested postoperative chemotherapy for high risk subsets of patients. Laitinen et al. [13] in a study of 31 lung carcinoid tumors (21 TC, 10 AC) reported a higher Ki-67 index in atypical versus typical carcinoid (3.8% versus <1%) with highest percentage of Ki-67 positive nuclei found in 2 atypical carcinoids (10–20%). In addition Ki-67 was positively correlated with apoptotic index (*P* < 0.01) in this study. The author concluded that the proliferation rate in carcinoid tumors is generally very low but increased in AC and can be related to worse prognosis. Arbiser et al. [14] studied 20 neuroendocrine lung tumors (5 TCs, 5 ACs, 5 LCNEC, and 5 SCC) in order to determine whether angiogenesis and proliferation rate (assessed by manually counting the number of Ki-67 positive cells in 1000 cells) correlate with tumor types. They found that the proliferation rate was significantly different between SCC and CT (*P* < 0.05); however, proliferation rate did not distinguish between TC and AC, nor between SCC and LCNEC. They concluded that increased rates of proliferation, but not angiogenesis, correlate with tumor type. Igarashi et al. [16] in a study of 18 lung carcinoid tumors (13 TCs, 5 ACs) reported a higher Ki-67 index in atypical versus typical carcinoid (8.6% versus 1.3%.) by counting 1000 tumor cells manually and this finding was statistically significant (*P* < 0.01). He also reported a close correlation between Cyclin B1 and Ki-67 expression in these tumors. Pelosi et al. [15], in a study of 7 resected lung carcinoids (2 TCs, 5 ACs), noted that proliferation activity, as assessed by Ki-67 labeling index, was generally low in LCTs (1% for TC, 1–17% for AC). The same authors further investigated 136 resected lung carcinoids (100 TCs, 36 ACs) and reported that ACs have a higher expression of Ki-67 labeling compared to TCs (mean 9% versus 2.3%). The methodology, manual versus image analyzer, the number of cells counted, and information on patient outcome are not available (unpublished observation). Walts et al. [19] in a study of 101 carcinoid tumours (78 TCs, 23 ACs) confirmed that mean Ki-67 indices are significantly different in typical and atypical pulmonary carcinoid tumors but show a considerable overlap in the distribution of values in both groups of tumors. In a recent review paper on neuroendocrine tumors of the lung, based on a number of previous published papers which included biopsy and resected specimens, Rekhtman [17] reported a weighted Ki-67 index mean value of 1.5% (range 0–2.3%) for TC and 7.7% (range 0–17%) for AC compared to 64% (25–96%) for small-cell lung carcinoma and 46% (20–90%) for LCNEC. In another recent systematic review paper, Pelosi et al. [18], using a question-answer methodology, concluded that Ki-67 is a feasible and potentially meaningful marker in lung NE tumours, but more data are needed to determine its ideal function in this setting of tumours. It has also been pointed out by these authors that a diagnostic role is currently lacking even though there are significant differences between TC and AC in most studies. The prognostic role of Ki-67 is debated, likely due to methodological and biological reasons and lack of widely agreed–upon cutoff thresholds. Zahel et al. [21] in a study of 200 lung carcinoids (114 TCs, 86 ACs) applied the grading system for gastrointestinal NE tumours to pulmonary carcinoids. In this study, compared to mitotic count, Ki-67 index showed a more significant correlation with advanced tumour stage and distant metastasis; in addition Ki-67 correlated significantly with larger tumour size and a positive nodal status. Rindi et al. [20] in a recent study of 399 pulmonary NE tumours (113 TCs, 84 ACs) reported an innovative evidence based proposal of a three-tire morphology independent grading system for NE lung tumours. Expert eye method, Aperio automated computer assisted quantitative method, and computer assisted manual method were used for Ki-67 counting. The Authors generated a three-tier grading system based on Ki67 index, mitotic count, and necrosis with lung specific cutoff thresholds, which provides an effective tool for accurately predicting prognosis and biological aggressiveness.

Although variable methodology has been used by above authors in counting Ki-67, it is clear that most of the studies observed a higher Ki-67 index in AC compared to TC although cutoff thresholds are variable in these studies. Our study is unique in that we utilized 2 counting techniques, namely, CIAM counting 5000 cells and MCM counting 2000 cells, to calculate Ki-67 index. Although time-consuming, we believe that image analysis allows for more objective, reproducible, and precise counts. Although we observed a strong correlation between two counting methods, it is important to note that Ki-67 labeling index calculated by MCM was slightly higher than that by CIAM for individual cases. This is attributed to the fact that MCM uses human eye for scanning hotspot areas compared to the precise CIAM tool, resulting in slightly imprecise estimation of the total number of cells in the background. In addition we counted a relatively larger number of cells (5000) by CIAM to reduce the margin of error and used statistical analysis to determine the minimum number of cells to be counted by CIAM. However, results of our study suggest that, in every day surgical pathology practice when Ki-67 index needs to be calculated, MCM may be used as a substitute. In such instances we recommend manual counting of at least 2000 cells in hotspot areas.

In conclusion, this study confirms that mitoses ≥2 is a powerful predictor of metastasis in LCTs. In addition, in this study, mean Ki-67 index is higher in AC compared to TC and in metastatic group compared to nonmetastatic group. Although this is a small sample size, there is suggestion that analysis of Ki-67 index along with mitoses and tumor size may be a useful adjunct for predicting metastasis and for initiating adjuvant multimodal therapy in lung carcinoid tumours. However a standardized methodology is needed to validate limits of Ki-67 index for practical prognostic applications and this would require larger prospective studies and testing on both small biopsies and resected specimens.

## Figures and Tables

**Table 1:** Correlation between Ki-67 index calculation methods and type of carcinoid and metastasis.

	AC (%)/TC (%)	MG (%)/NMG (%)
	(*P* value)	(*P *value)
Ki-67 (MCM)	2.32/1.37	2.10/1.39
	(0.71)	(0.239)

Ki-67 (CIAM)	0.95/0.72	1.01/0.71
	(0.299)	(0.281)

AC: atypical carcinoid; TC: typical carcinoid; MG: metastatic group; NMG: nonmetastatic group; MCM: manual conventional method; CIAM: computer image analysis method.

**Table 2:** Relationship between various clinical and pathologic factors and metastasis.

	Metastasis		Sensitivity	Specificity	OR	*P* ^Ψ^
	Absent (*n* = 41)	Present (*n* = 7)	(%)	(%)	(95% CI)	
Carcinoid type						
Typical^*γ*^	34	3	57.1	82.9	6.48	0.039
Atypical	7	4			(1.18, 35.58)	
Size, cm						
<3^*γ*^	29	2	71.4	70.7	6.04	0.080
≥3	12	5			(1.03, 35.54)	
Mitosis						
<2.00^*γ*^	36	3	57.1	87.8	9.60	0.017
≥2.00	5	4			(1.64, 56.09)	
Ki67 (MCM)						
<0.50^*γ*^	13	0	100.0	31.7	—	0.166
≥0.50	28	7				
Ki67 (CIAM)						
<0.50^*γ*^	21	1	85.7	51.2	0.16	0.106
≥0.50	20	6			(0.02, 1.44)	
Mitosis ≥2 and Ki67 (CIAM) ≥0.50						
No^*γ*^	38	4	42.9	92.7	9.50	0.033
Yes	3	3			(1.42, 63.72)	
Size ≥3 and Ki67 (CIAM) ≥0.50						
No^*γ*^	34	3	57.1	82.9	6.48	0.039
Yes	7	4			(1.18, 35.58)	
Mitosis ≥2 and size ≥3						
No^*γ*^	39	4	42.9	95.1	14.62	0.018
Yes	2	3			(1.86, 115.19)	

MCM: manual conventional method; CIAM: computer image analysis method; OR: odd ratio; CI: confidence interval.

^*γ*^The reference category for calculation of sensitivity, specificity, and odd ratios.

^Ψ^Statistical comparisons made using Fisher's exact two-tailed test.

**Table 3:** Ki-67 index: correlation between CIAM and MCM.

Method	Ki-67 index	Pearson correlation coefficient
	Mean (SD)	*r* (*P* value)
CIAM	0.77 (0.68)	0.929 (0.001)
MCM	1.6 (1.5)	

*r* > 0.8 indicates strong correlation; *P* value <0.05, significant; CIAM: computer image analysis method; MCM: manual conventional method; SD: standard deviation.

**Table 4:** A summary of select reported Ki-67 labeling indices in lung carcinoid tumors.

Studies	Number of cases	% of Ki-67 positive cells, mean (range)		Counting method
		TC	AC	
Costes et al. 1995 [12]	47	0.45 (0–3.04)	2.43 (0–6.05)	CIAM
Arbiser et al. 2001 [14]	10	1	9	MCM
Igarashi et al. 2004 [16]	18	1.3 (0.3–2.3)	8.6 (0.2–17)	MCM
Pelosi et al. 2005 [15]	7	0.5 (0-1)	7.2 (1–17)	NS
Walts et al. 2012 [19]	101	3.7	18.8	CIAM
Zahel et al. 2012 [21]	200	1.8	3.7	MCM
Rindi et al. 2014 [20]	197	<4	4–<25	MCM, AACAM, and CAMM
Present case	48	0.72	0.95	MCM and CIAM

CIAM indicates computer image analysis method; MCM: manual conventional method; NS: not specified; AACAM: Aperio automated computer assisted method; CAMM: computer assisted manual method; TC: typical carcinoid; AC: atypical carcinoid.
